# Expression of mRNA for glutamate receptor subunits distinguishes the major classes of retinal neurons, but is less specific for individual cell types

**Published:** 2007-06-18

**Authors:** Tatjana C. Jakobs, Yixin Ben, Richard H. Masland

**Affiliations:** Massachusetts General Hospital, Harvard Medical School, Boston, MA

## Abstract

**Purpose:**

To investigate the expression of ionotropic glutamate receptor subunits by retinal neurons, to assess the extent to which different functional types of retinal neurons are characterized by the expression of the receptor subtypes.

**Methods:**

Rod photoreceptor cells and bipolar cells were identified in retina dissociates. Amacrine cells were identified in dissociates from transgenic mice or by staining with an antibody against the extracellular carbohydrate epitope CD15. Ganglion cells were identified by retrograde axonal transport of FITC-dextran or by green fluorescent protein (GFP) fluorescence in a transgenic strain. We examined the receptors simultaneously using non-quantitative single-cell reverse transcriptase polymerase chain reaction for GluR1-R4 (α-amino-3-hydroxy-5-methyl-4-isoxazole propionate (AMPA) receptors), GluR5-R7, and KA1 and 2 (kainate receptors), δ1 and δ2 subunits, and the N-methyl-D-aspartate (NMDA) receptor subunits NR1, 2a-d, and 3a.

**Results:**

The expression of glutamate receptors on bipolar cells and rod photoreceptors was limited: Neither expressed functional NMDA receptors, and rods were also negative for AMPA receptors. The sample of ganglion cells included examples of many ganglion cell types; these were distinguished morphologically using quantitative parameters defined in a previous cluster analysis. All types of ionotropic glutamate receptors were found to be expressed on ganglion cells. The iGluR subunits GluR4, KA2, δ1, and NR1 were expressed on almost all ganglion cells examined.

**Conclusions:**

Despite the heterogeneity of ganglion cell types, differences among them in this PCR-based method were minor. Thus, retinal interneurons are characterized by expression of distinctive glutamate receptor types, but functional differences among ganglion cells seem to be reflected instead in the amounts as well as spatial distributions of a widely expressed group of receptors.

## Introduction

Molecular markers for the diverse neuronal types of the central nervous system remain elusive. Here, we report a study distinguishing, in the retina, the two major categories of neurons: interneurons, which are small in volume and project locally, and projection neurons, which have long axons and collect information within a large dendritic arbor. In the retina, photoreceptors, horizontal, bipolar and amacrine cells make up the former, and ganglion cells the latter group [1-3]. None of these is a homogeneous population. For example, there are 12-15 types of ganglion cells in the mammalian retina; they can be distinguished by their size, stratification within the inner plexiform layer (IPL), and various subtler morphological features [4-7]. These morphological types serve different functional roles, corresponding to different parallel channels of information transfer [2,8]. For the majority of ganglion cell types, the correlation between morphology, and function has not yet been achieved, nor is much known about the molecular mechanisms that generate functional differences between ganglion cell types.

In this study we examined the repertoire of ionotropic glutamate receptors expressed by several types of retinal neurons. Ionotropic glutamate receptors can be subdivided into three classes depending on their preference for the glutamatergic agonists a-amino-3-hydroxy-5-methyl-4-isoxazole propionate (AMPA), kainate, and N-methyl-D-aspartate (NMDA). Characteristic subunit compositions are the molecular basis of these pharmacological differences [9-12]. α-amino-3-hydroxy-5-methyl-4-isoxazole propionate receptors (AMPARs) are thought to underlie the ganglion cell response to excitation by glutamate released at the bipolar cells' ribbon synapses [13,14], but kainate and NMDA receptors have been detected on ganglion cells and a variety of other cell classes in the retina [15].

Existing methods for receptor localization have particular strengths and weaknesses. Though in-situ hybridization allows identification of a cell's layer (and thus a clue to its cell class), it does not reveal the cell's morphology. In contrast, immunocytochemical staining of neurotransmitter receptors leads to a punctate labeling in the synaptic layers; since the signals arise from overlapping populations of cells, it is difficult to identify individual cells [16,17]. Single-cell PCR is a very sensitive method but requires some means of prior identification of the cell studied. In this study we have imaged individual cells' morphologies before microdissection and used a multiplex RT-PCR reaction [18,19] for the simultaneous amplification of all subtypes of AMPA, kainate and NMDA receptors, the non-ligand binding subunits δ1 and δ2, and positive (β-actin) and negative (rod-opsin) internal controls that were always included. We did not attempt to establish quantitative differences in gene expression levels, as this would be of questionable reliability on the level of single cells of different types and vastly different sizes. Therefore we made a binary distinction for all genes in our sample as either detectable or undetectable. Still, there were readily demonstrable differences in the panels of glutamate receptors expressed by three major retinal cell classes: rod photoreceptors, bipolar cells, and ganglion cells. Within the individual members of the classes of bipolar and ganglion cells, however, the differences are much less striking and functional differences may be more related to the amounts or localization of the different glutamate receptors than to their simple presence or absence. In other words, most bipolar and most ganglion cells may express more or less the same set of receptors, but employ them differentially.

## Methods

### Isolation of single ganglion cells from mouse retina

Light adapted 5-8 week old C57bl, YFP-12, line 357 [20], and GFP-M [21] mice were used for all experiments. The mice were anesthetized with isoflurane and killed by an intraperitoneal injection of 100 mg/kg ketamine (Hospira, Lake Forest, IL) and 20 mg/kg xylazine (Lloyd, Shenandoah, IA). The eyes were removed and hemisected along the ora serrata. The retinas were taken out of the eyecup, cut into 4-6 pieces and stored in oxygenated Ames' medium (Sigma, St. Louis, MO) until further use. All protocols were in accordance with the rules of the Subcommittee on Research Animal Care of the Massachusetts General Hospital.

To identify generic ganglion cells (without imaging their morphology), retrograde dye labeling was used. The mice were anesthetized, and small craniotomy holes were bored above the superior colliculi on both sides. About 0.5 ml of FITC-dextran (MW 3000, Invitrogen, Carlsbad, CA) was injected into the superior colliculus using a glass microcapillary connected to a Picospritzer II (General Valve Corporation, Fairfield, NJ). The craniotomy holes were filled with Gelfoam (Upjohn, Kalamazoo, MI), and the skin was closed with surgical clips. Two days after the procedure, the mice were sacrificed as described above. In some cases, we used mice of the YFP-12 line, which expresses YFP under the nominal control of the Thy1 promoter, resulting in a bright expression of YFP in ganglion cells and other cells of the inner retina [21]. Though many different types of cells are labeled, the intense fluorescence together with the size of the cell body permits one to identify at least the largest ganglion cells in the population. In either case, pieces of retina were dissociated for 20 min in 4 ml Hank's balanced salt solution (HBSS, Invitrogen) with 0.5 mg/ml papain (Worthington, Lakewood, NJ) at 37 °C. The tissue was centrifuged briefly at low speed, and the papain solution was exchanged for HBSS containing 10% horse serum and 200 U/ml DNAseI (Sigma). The tissue was then triturated with a heat-polished glass Pasteur pipette to break up remaining clumps, and the resulting cell suspension was used to pick up single cells.

In addition to ganglion cells, single rod photoreceptors and bipolar cells were identified by their morphology in the dissociate and collected in the same way. Amacrine cells cannot unambiguously be identified by soma morphology alone. However in the GFP-M mouse line, some of the GFP-labeled cells are amacrine cells of various subtypes. Amacrine cells microdissected from GFP-M retinas (see below) were picked up and analyzed in the same way as ganglion cells. In addition, anti CD15 antibodies are known to label two types of amacrine cells in the mouse retina [19]. FITC-conjugated anti-CD15 antibodies (Beckton Dickinson, San Diego, CA) were directly diluted 1:25 into suspensions of dissociated cells from C57bl/6 retinas. This antibody recognizes an extracellular epitope and leads to staining of the cell membrane CD15+ amacrine cells. These labeled amacrine cells were collected for analysis. Though bipolar cells can be recognized as bipolar cells in the dissociate, it is usually difficult to make any further distinction on the basis of morphology alone. In some experiments we therefore used the mouse line 357, which expresses YFP under the control of the gustducin promoter. Fortuitously, this resulted in the labeling of one type of ON cone bipolar cell, and a dim labeling of rod bipolar cells in the retina [20,22,23]. We collected rod bipolar cells, GFP-labeled cone bipolar cells, and other, unlabeled bipolar cells from dissociates of these retinas, as well.

In some cases, we imaged individual ganglion cells before collection for RT-PCR. For this purpose, retinas from the GFP-M cell line were used. These mice express GFP under the control of the Thy1 promoter [21] but contain only about 30 GFP-labeled ganglion cells (of various types) per retina. This permits imaging of individual ganglion cells. Retinas from GFP-M mice were prepared as described above. Then, small pieces of retina were cut and mounted on 3 μm Isopore filters (Millipore, Bedford, MA), and evaluated at 20x magnification on a Zeiss Axioscope 200 (Carl Zeiss Inc., Thornwood, NY). Individual ganglion cells were chosen for isolation and photographed with a cooled CCD camera (Spot Camera, Diagnostic Instruments, or Cool Snap, Roper Scientific, Ottobrunn, Germany). The piece was transferred to a Zeiss Axiovert 200 and the chosen cell was excised in a small piece of retina (approximately 100x100 μm) under microscopic control using a scalpel blade mounted on a micromanipulator (Newport, Irvine, CA). The piece of tissue was then transferred to an Eppendorf tube containing 50 μl HBSS with 0.5 mg/ml Papain and incubated at 37 °C for 5 min. The digestion reaction was quenched with 50 μl HBSS containing 10% horse serum and 200 U/ml DNAseI. Small clumps of tissue were triturated with a pipette tip. The mixture was pipeted into one etched ring of a Gold Seal Fluorescent Antibody microscope slide (Becton Dickinson) and allowed to settle for 1 min. In about half of the cases, the GFP-expressing cell had survived the procedure and could be clearly identified amongst the other unlabeled cells.

### Collection of single cells for reverse transcriptase polymerase chain reaction

Microcapillaries for collection of single cells were pulled from silanized 1.5 mm glass blanks (World Precision Instruments, Sarasota, FL) on an electrode puller (Narishige Tokyo, Japan). The labeled cell was aspirated, washed in Ringer's solution with 0.5% bovine serum albumin (BSA; Sigma, St. Louis, MO), aspirated again with a fresh microcapillary and placed into a thin-wall reaction tube (Applied Biosystems, Foster City, CA) containing 5 μl of reverse transcriptase polymerase chain reaction (RT-PCR) reaction buffer with 2.75 pmol each of all first round polymerase chain reaction (PCR) primers. For every cell, about 100 nl of the washing solution were aspirated as a negative control. As additional controls, neurons other than ganglion cells were also collected. Bipolar cells are easily distinguished by their morphology even after dissociation, as their dendrites and axon withstand the procedure well. Intact rod photoreceptors are also encountered. Total RNA from whole retina (100 ng), or 1000-2000 cells from a freshly dissociated retina, served as positive controls.

### Reverse transcription and polymerase chain reaction

For reverse transcription and the first round PCR the Access PCR kit (Promega, Madison, WI) was used. The ganglion cells were lysed at 65 °C for 1 min, and reverse transcriptase (2 U) and Tfl polymerase (2 U) were added to yield final volume of 10 μl. Reaction conditions for the RT-PCR were: 45 min at 48 °C for reverse transcription, followed by 20 cycles 1 min at 94 °C, 1 min at 60 °C, 2 min at 68 °C, and a final extension step of 7 min at 68 °C. Second round PCRs were done separately for each probe using 1/60 of the first round product, 7.5 pmol each of the inner primers, and AmpliTaq Gold DNA polymerase (Applied Biosystems, Foster City, CA). Negative controls were always run in parallel with the cells. Primer sequences and the lengths of the expected amplicons are given in Table 1. In the case of the AMPA receptors, the outer primers, used in the first round PCR amplify all four receptor subtypes simultaneously, the second PCR, using nested primers, distinguishes between individual subtypes and the flip- and flop isoforms. For the kainate receptor and the delta subunits, too, nested primers were used in the second round PCR. All primer combinations spanned at least one intron/exon boundary. The primers for the NMDA receptors and actin were used as described by Paarmann et al. [24], with the exception that we designed nested primers for NR2b and NR2c. Primers for metabotropic glutamate receptor 6 (mGluR6) used in some experiments were described by Maxeiner et al. [25]. All of the second-round reaction mix was loaded on a 2% agarose gel, electrophoresed, and photographed. After electrophoresis, some amplification products were chosen for confirmatory restriction enzyme digestions. The DNA was eluted from the gel using the Qiaex II gel extraction kit (Qiagen, Hilden, Germany) and cut with the appropriate restriction enzyme (Table 1). Probes were prepared for sequencing in the same manner. Sequencing was performed with the inner PCR primer on an automated sequencer (DNA sequencing core facility of Massachusetts General Hospital). The resulting sequences were compared to the nucleotide database using the BLASTn algorithm at the website of the National Center for Biotechnology Information.

**Table 1:** Primers used for amplification of cDNA of the glutamate receptor isoforms, actin, rod-opsin, mGluR6, and melanopsin.

**Amplicon**	**Sequences**	**bp**
GluRl flip	up: tgggtgcctttatgcagcaaggatg, low: gtccgtatggcttcattgatggattgc up: aatlgcttatggcacattggaagca. low: tcttgtccttacttccggagtcctt	419
GluRl flop	up: tgggtgcctttatgcagcaaggatg. low: gtccgtatggcttcattgatggattgc up: aattgcttatggcacattggaagca. low: gtcttgtccttggagtcacctccccc	419
GluR2 flip	up:: tgggtgcctttatgcagcaaggatg. low: gtaacagaactctatcaaagccacca up: ataaaatgtggacttatatgaggag. low: tcttgtccttacttccggagtcctt	349
GluR2 flop	up: : tgggtgcctttatgcagcaaggatg. low: gtaacagaactctatcaaagccacca up: ataaaatgtggacttatatgaggag, low: gtcttgtccttggagtcacctccccc	349
GluRJ flip	up: : tgggtgcctttatgcagcaaggatg, low: gtaacagaactctatcaaagccacca up: ccccatagagagcgctgaagatt. low: tcttetccttacttccggagtcctt	458
GluR3 flop	up: : tgggtgcctttatgcagcaaggatg, low: gtaacagaactctatcaaagccacca up: ccccatagagagcgctgaagatt. low: gtctmtccttggagtcacctccccc	458
GluR4 flip	up:: tgggtgcctttatgcagcaaggatg, low: gtaacagaactctatcaaagccacca up: attgcctatggaacacttgattcg. low: tcttgtccttacttccggagtcctt	419
GluR4 flop	up: : tgggtgcctttatgcagcaaggatg. low: gtaacagaactctatcaaagccacca up: attgcctatggaacacttgattcg. low: gtcttgtccttggagtcacctccccc	419
KA1	up: ggctccaccatgaccttcttcca. low: tcccaccacttgcgcttcag up: gaftgccagagtgttgaattccaat. low: atttccagccgnttgttctcctg	207
KA2	up: ggctccaccatgaccttcttcca. low: tcccaccacttgcgcttcag up: aatcgcccgcgtcctcaactcccgc, low: atttccagccggttgttctcctg	207
GluR5	up: ccctgactcagacgtggtggaa, low: agaaggtcattgtcgagccatctc up: gttggagctctcatgcagcaagg. low: agaaggtcattgtcgagccatctc	358
GluR6	up: ccctgactcagacgtggtggaa. low: ctatcttggtttgcttagctaaa up: gttggagctctcatgcagcaagg. low: ctatcttggtttgcttagctaaa	196
GluR7	up: ccttacgagtggtatgatgctc. low: atgaaggcccacatcttctcaa up: tctggtttggaatgggctccctg. low: atggtggccccgtccttgcacgca	238
delta 1	up: acagccaaccttgccgctttcctcac. low: ccataactctagagcagccacgagg up: tctggcggaccataagcaaga. low: cataactctagagcagccacgag	439
delta 2	up: acagccaaccttgccgctttcctcac. low: atcctctttcgatgggacccgggag up: caggatctgtccaagcaaacaga. low: ctcctcccttctgctttgcat	469
NR1^(1)^	up: gctgtacctgctggaccgct. low: gcagtgtaggaagccactatgqatc	219
NR2a^(1)^	up: gctacgggcagacagagaag. low: gtggttgtcatctggctcac	257
NR2b^(2)^	up: gctacaacacccacgagaagag. low: gagagggtccacgctttcc up: ccacgagaagaggatctaccag. low: caatgacaaacggtgcctcctc	284
NR2c^(2)^	up: aaccacaccttcagcagcg. low: gacttcttgcccttggtgag. up: atataaccccctacaccaagct. low: gttgtagctgacagggctgaaa	416
NR2d^(1)^	up: cgatggcgtctggaatgg. low: agatgaaaactgtgacggcg	265
NR3a^(1)^	up: ccgcgggatgccctactgttc. low: ccauttuttcatggtcaggat	417
actin^(1)^	up: gggaaatcgtgcgtgacatt. low: cggatgtcaacgtcacactt	255
rod-opsin	up: tacacactcaagcctgaggt. low: cctagtgggtgaauatgtatg	291
melanopsin	up: tcttcatcttcagggccatc. low: ttctctgctgtaggccacat up: tcaagagtccctgggttctg. low: ttagaccctgctacagatgtct	362
mGIuR6^(3)^	up: catggtcacgtgtacagtgtatgc. low: cttcttgaggctgcgcttccgc	298

For most genes two sets of nested primers were used. The length of the amplicon in base pairs refers to the inner polymerase chain reaction product. The primers for the NMDA receptor subtypes were as described by Paarmann [24], with the exception of the inner primers for NR2b and NR2c. The primers for mGluR6 were as described by Maxeiner [25].

### Preparation of cDNA from whole retina

Retinas from adult C57Bl were prepared as described above and total RNA was extracted using the RNeasy mini kit (Qiagen) with an on-column DNAse step according to the manufacturer's instructions. The total RNA was either converted to cDNA using the Superscript Preamplification system (Invitrogen) or used for RT-PCR directly.

### Cell measurement and classification

Three parameters were recorded for each cell chosen for single-cell RT-PCR analysis. The depth of stratification [1] was estimated before dissociation of the tissue by comparing the focal plane of the dendritic arbor with those of the inner nuclear layer (INL) and the ganglion cell layer (GCL) before dissociation of the tissue. Dendritic field area, defined as the smallest convex polygon around the cell's dendrites [2] and dendritic length per unit area [3] were calculated from the cell images as follows: The images were adjusted for contrast and median filtered in Adobe Photoshop 7 (Adobe Systems Inc. San Jose, CA) and then manually traced using the Neurolucida analysis software (Microbrightfield, Williston, VT) from the wide-field micrographs taken before dissociation of the retina. This resulted in 2D representations of the cells. Total dendritic length and dendritic field area were calculated using Neuroexplorer (Microbrightfield) and dendritic length per unit area was calculated. We compared the cells in our sample to a prior study of mouse ganglion cells, also using cells from the GFP-M mouse line [26], in which the cells were grouped into "types" by a formal cluster analysis. As the three parameters are different in units and range, each parameter was normalized individually (both here and in the previous study) using the cell population's means and standard deviations. The earlier study classified mouse ganglion cells into 11 clusters. The cells in this study were assigned to one of the 11 clusters by user-written software that for any cell minimized the Euclidean distance in the three dimensional parameter space to the cluster centers.

## Results

We first verified that all isoforms of ionotropic glutamate receptors together with internal controls (actin and rod-opsin) can be amplified simultaneously from total retina RNA in a single-tube RT reaction and first round PCR with gene-specific priming, followed by second round PCR reactions for all gene-specific primer pairs separately. We found that a combination of a relatively short first round PCR (19 cycles) and a longer (30-32 cycles) second round PCR with nested primers was effective in amplifying all iGluR isoforms from 30-100 pg total retina RNA (data not shown). The identity of all PCR products was verified by sequencing.

### Adaptation of iGluR reverse transcriptase polymerase chain reaction to single-cell format

**Figure 1:**
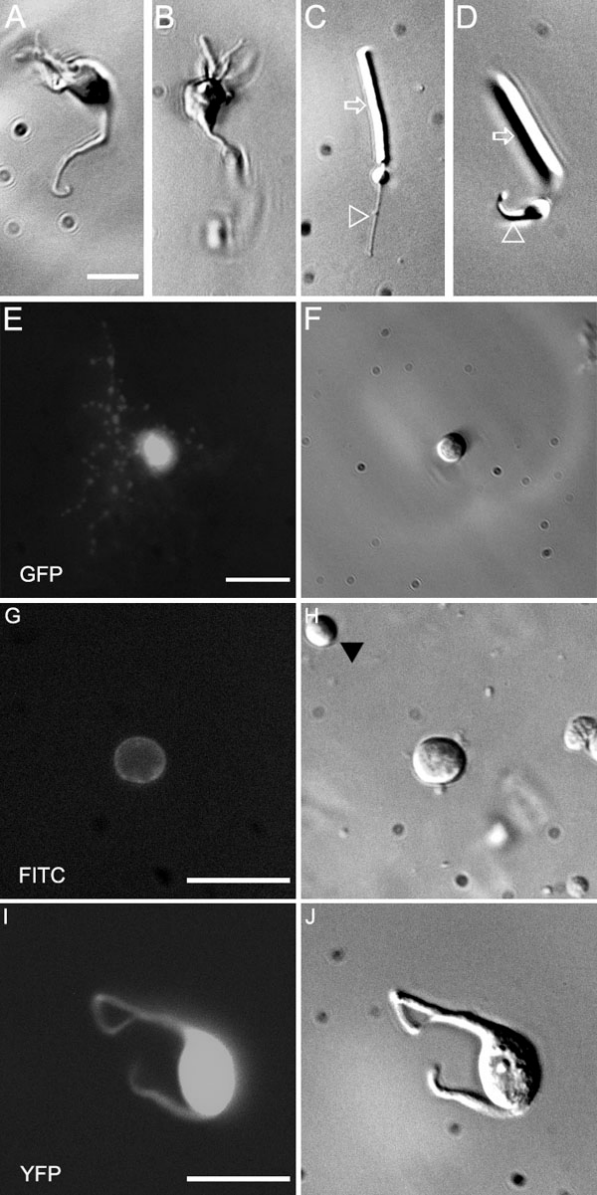
Isolation of retinal interneurons. **A**, **B**: Bipolar cells, easily recognizable by their morphology. **C**, **D**: Rod photoreceptors. Note the well-preserved morphology of the rod outer segment (arrows) and the axon (arrowheads). Scale bar for **A**-**D** reprsents 10 μm. **E**: Amacrine cell from a GFP-M retina before dissociation. The focus is on the dendrites of this small-field cell. **F**: The same cell after dissociation of the retinal piece. Scale bar for **E** and **F** represents 20 μm. **G**: CD15 amacrine cells can be directly visualized in the dissociate from C57BL retina by addition of a FITC-labeled anti-CD15 antibody. The immunofluorescence delineates the cell membrane because CD15 is an extracellular glycoprotein epitope. **H**: the same cell in Hoffman optics. Note that the other cell body (arrowhead) is negative for CD15. Scale bar for **G** and **H** 20 μm. **I**: Immature ganglion cell at P5 from an YFP-12 mouse. **J**: The same cell in Hoffman optics. Scale bar for **I** and **J** represents 20 μm.

**Figure 2:**
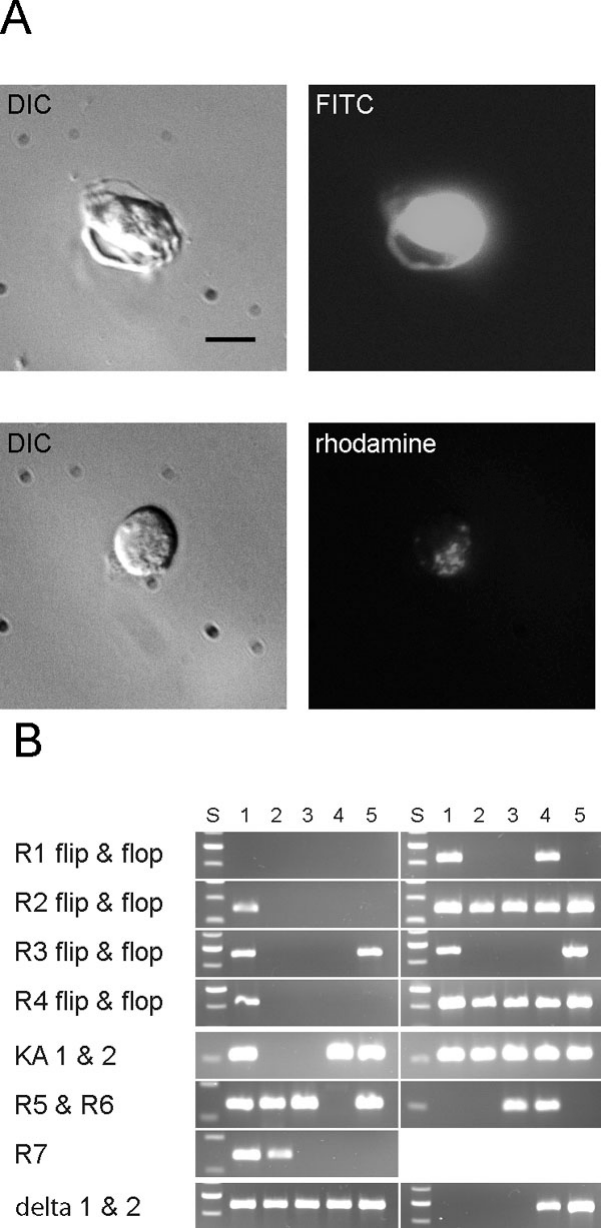
Isolation and reverse transcriptase polymerase chain reaction of ganglion cells. **A**: Ganglion cells isolated from mouse strain YFP-12, identified by their bright fluorescence (upper panels) and from C57BL identified by fluorescent inclusion bodies in the cytoplasm 2 days after injection of rhodamine-dextran into the superior colliculus (lower panels). Scale bar represents 10 μm. **B**: Single ganglion cells were assayed for all AMPA receptors in flip and flop isoforms, kainate receptors KA1&2 and GluR5-7, δ1 and δ2, NMDA receptors NR1, NR2a-d, and NR3a, and actin and rod-opsin as positive and negative internal controls. S= molecular weight standard (100 bp ladder), results from 5 individual ganglion cells are shown.

We then applied this RT-PCR protocol to single cells isolated from freshly dissociated mouse retina (see Figure 1 and Figure 2A for examples of dissociated retinal neurons). RT-PCR is vulnerable to contamination through carry-over of cell debris, mRNA that has been released from lysed cells, or the inadvertent collection of more than one cell. The following precautions were aimed at avoiding these sources of contamination. Only cells with smooth cell membrane and no signs of incipient cell lysis were chosen for collection. Every cell was picked with a glass capillary from the dissociation mix, transferred to another well with fresh medium for washing, and re-picked with a new capillary before transfer to the reaction tube. Controls from the washing medium were taken from every cell. Finally, every cell was assayed for rod-opsin in parallel to the iGluRs (Figure 2B). Rods are by far the most common cell type in the mouse retina, and a contamination of the cell chosen for analysis by debris or external RNA is likely to lead a positive test for rod-opsin.

### The expression of ionotropic glutamate receptor subunits in bipolar cells, rods, and amacrine cells

**Figure 3:**
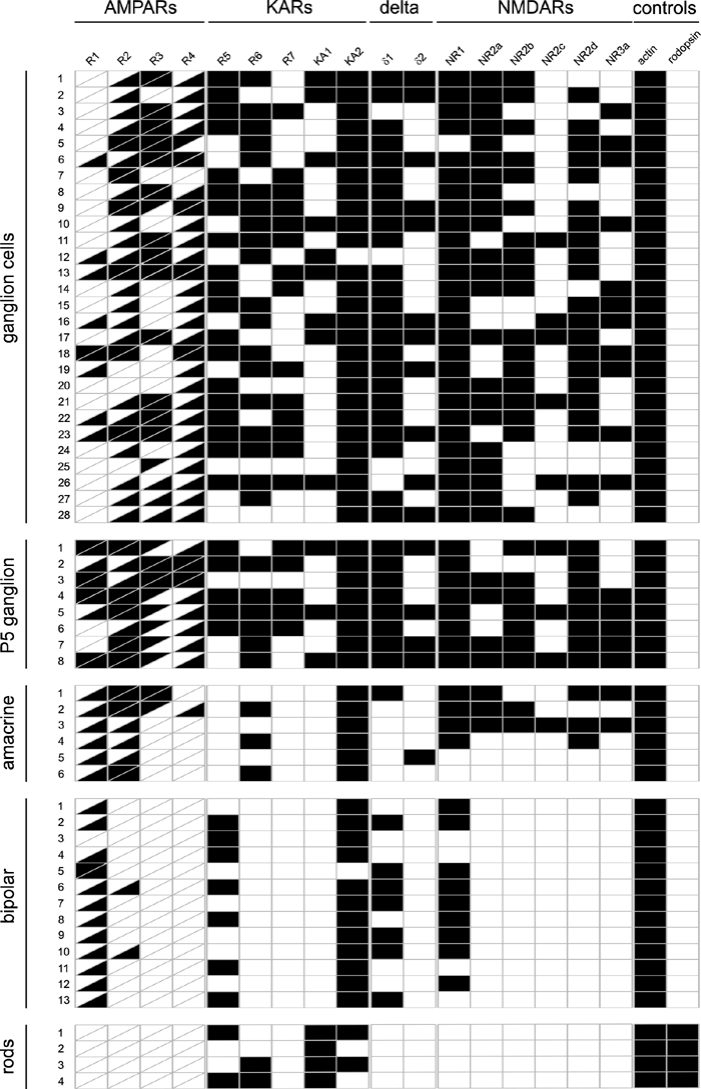
Ionotropic glutamate receptor expression profile in adult mouse retina. Ganglion cells, immature ganglion cells (P5), amacrine cells, bipolar cells, and rod photoreceptors were tested. Black rectangles indicate a positive polymerase chain reaction result. The rectangles for the AMPA receptors GluR1-4 are divided to indicate expression of the flip (upper left) and the flop (lower right) isoforms of these receptors.

Bipolar cells can easily be recognized and distinguished from other retinal neurons by their morphology after dissociation (Figure 1A,B). There are morphological differences between rod- and cone bipolar cells; rod bipolar cells have rounder and bulkier axon terminals than cone bipolar cells, and the dendrites emerge as a tuft from the cell body. We selected cells of cone bipolar cell morphology, but it is not possible to further discriminate cone bipolar cell subtypes after dissociation. The bipolar cells (n=13) in our sample displayed a signature of ionotropic glutamate receptor (iGluR) expression that was obviously different from that in the ganglion cells. First, though some bipolar cells were found to express AMPA receptors, these were always of the subtypes GluR1 and GluR2. As expected, some bipolar cells were positive for kainate receptor subunits, usually KA2 and GluR5. Though some of the bipolar cells in our sample were positive for NMDA receptor 1 (NR1), none would be expected to express functional NMDA receptors, as the other subunits were always absent. Finally, in no case did we observe expression of δ2 (Figure 3).

**Figure 4:**
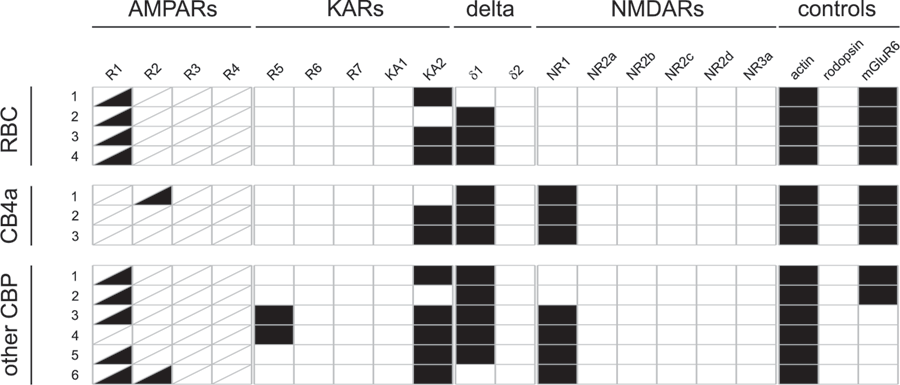
Ionotropic glutamate receptor expression in mouse bipolar cells. Rod bipolar cells (RBC), YFP^+^ CB4a cone bipolar cells from transgenic mouse line 357, and other, YFP^-^ bipolar cells from the same mouse line were tested. Black rectangles indicate a positive PCR result. The rectangles for the AMPA receptors GluR1-4 are divided to indicate expression of the flip (upper left) and the flop (lower right) isoforms of these receptors.

The main focus of this study was on ganglion cells, so we did not initially attempt to distinguish between types of bipolar cells. We did ask, however, whether there were any obvious differences between rod- and cone bipolar cells and ON- and OFF- cone bipolar cells. For this purpose, we collected bipolar cells from dissociates of a transgenic mouse strain (number 357) which expresses YFP in one type of ON cone bipolar cell (CB4a), and -less brightly- in rod bipolar cells [23]. These bipolar cells were also probed for mGluR6 expression. Rod bipolar cells (n=4) were found positive for GluR1, KA2, and δ1 in addition to mGluR6. CB4a cells (n=3) expressed KA2, δ1, and NR1 in addition to mGluR6, GluR1 was not found in these cells, though one of them was positive for GluR2. We also collected YFP-negative bipolar cells (n=6), four of which were negative for mGluR6, and therefore most likely OFF bipolar cells. The RT-PCR results are summarized in Figure 4. We did observe bipolar cells that express mRNA for AMPA receptors (usually GluR1) and kainate receptors (GluR5 and KA2) together. This is surprising, as OFF bipolar cells have been described to use either AMPA or kainate receptors at the cone/bipolar cell synapse [27]. We cannot decide on the basis of our PCR data, whether GluR1 is expressed in the bipolar cell dendrites in these cells, or perhaps as an autoreceptor at the axon terminal.

Rod photoreceptors can also be distinguished by their morphology after dissociation (Figure 1C,D). Single rods (n=4) were collected and assayed for their iGluR expression. We never observed either AMPA or NMDA receptor subtypes or δ1/2, however rods did test positive for kainate receptors. Interestingly, they were the only cell type in our sample that invariably expressed KA1. Expression of GluR5, GluR6, and KA2 was also found in two cases each.

Amacrine cells are not as easy to recognize on morphological criteria after dissociation, but the mouse line GFP-M usually contains several labeled amacrine cells per retina. We isolated three small-field amacrine cells from a GFP-M retina (Figure 1E,F). In addition, we isolated three amacrine cells staining positive with an antibody against the CD15 epitope (Figure 1G,H), which has been shown to label two types of wide-field amacrine cells in mice [19]. All amacrine cells in our sample were positive for AMPA receptors, always expressing GluR1 and GluR2, and more rarely GluR3 and GluR4 (2/6 and 1/6). All of the amacrine cells in our sample expressed KA2, and 3/6 also were positive for GluR6. Finally, 4/6 amacrine cells expressed NMDA receptors, and 2/6 were negative. Both of the negative cells were wide-field cells.

This leaves two classes of retinal neurons uncovered: cone photoreceptors and horizontal cells. Both of these are rare in the mouse retina, and cones seem to be particularly fragile, as we did not encounter them often enough to collect them for analysis. Horizontal cells cannot be distinguished from other large cell bodies after dissociation, because they lose axon and dendrites. Unfortunately, only intracellular epitopes are available to stain horizontal cells, making it necessary to use detergents to permeabilize the cells. This renders the cell too fragile to be picked up and transferred for analysis. Cones and horizontal cells were therefore not included in our sample.

### All ganglion cells express AMPA, kainate, and NMDA receptors

After dissociation of the retina into single cells, ganglion cells lose their dendrites and can no longer be recognized with certainty (Figure 2A). We therefore used retrograde labeling with FITC-dextran to label them prior to the experiment. The fluorescent granules are still visible in the cell body after dissociation and allow for unambiguous identification of ganglion cells. In some cases we also used dissociated retinas from YFP-12 mice; in these retinas there is bright expression of YFP in about a third of the ganglion cells [21] (Figure 2A). Single ganglion cells were aspirated, but without any further attempt at identifying cell types. Typical results of single-cell RT-PCR on isolated ganglion cells are shown in Figure 2B.

In our initial sample of 28 generic ganglion cells, we always found the expression of at least two subtypes of AMPA receptors (Figure 3). GluR4 was almost invariably expressed (27/28), GluR2 and GluR3 were found frequently (25/28 and 18/28) whereas GluR1 is more rare (9/28). The combination encountered most commonly was GluR2/R3/R4 (12/28), followed by GluR2/R4 (6/28), and GluR1/R2/R3/R4 (5/28). For almost all receptor subtypes, the flop isoforms dominated; an exception was GluR3, where both isoforms were expressed with similar frequency (see Figure 3 for a summary of the RT-PCR results).

Kainate receptors were also found on ganglion cells. The KA2 subunit was encountered in 27/28 cells, whereas KA1 was found only in 9/28 cells. As most ganglion cells in our sample also expressed GluR5 or GluR6 (19/28 for both isoforms), GluR7 (14/28) or combinations of them, all but 2 cells would be expected to express functional, high-affinity kainate receptors.

A similar result was obtained for the NMDA receptors. NR1, the common subunit, was detectable in all but one cell, usually together with NR2a (22/28), NR2b (18/28), and NR2d (21/28), and more rarely, NR2c (5/28). Of the 28 cells 11 also expressed NR3a. The most common combination was NR1/2a/2d/3a.

In addition to these ionotropic glutamate receptors, the "orphan" subunits δ1 and δ2 have been found in the mammalian retina, in particular in S1 amacrine cells [16]. We found that the majority of ganglion cells (25/28) expressed δ1, and that δ2 was also fairly common (11/28). Taken together, these data suggest that almost all ganglion cells co-express all three types of ionotropic glutamate receptors.

**Figure 5:**
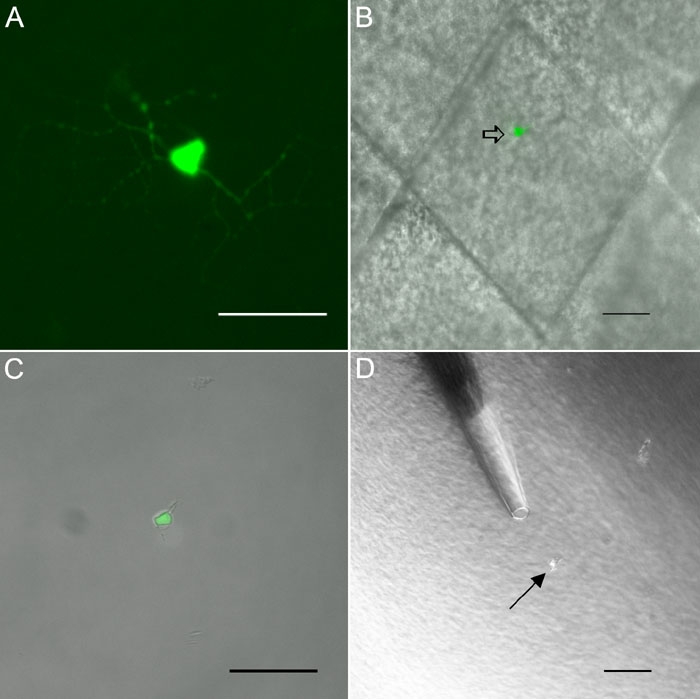
Microdissection procedure for morphologically characterized ganglion cells. **A**: GFP-labeled ganglion cell. Before isolation ganglion cells from GFP-M retinas were photographed and their level of stratification in the IPL was measured. **B**: a piece of tissue (about 100 mmx100 mm) containing only this one labeled ganglion cell was cut out. DIC and GFP images of the same piece are superimposed. Double arrow indicates the targeted ganglion cell. **C**: After dissociation the fluorescent ganglion cell was picked up from the cell suspension and transferred to another well with fresh buffer for washing. DIC and GFP pictures for the same isolated cell are superimposed. **D**: Finally the cell was transferred directly into the reaction tube containing PCR buffer. The transfer pipette is visible in the upper half of the picture, the arrow indicates the cell. All scale bars equal 50 μm.

**Figure 6:**
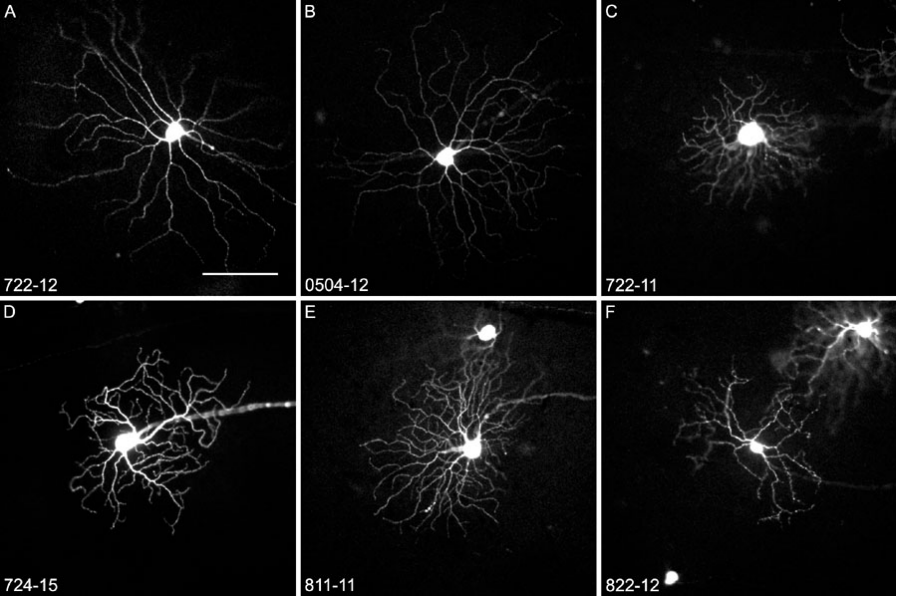
Gallery of ganglion cells taken for single cell analysis of their iGluR expression profile. **A**, **B** are large ON cells; **C** is small OFF cell; **D** is medium field diameter cell; **E** is bistratified cell; **F** is small OFF cell. Cell ID is indicated for every cell. Scale bar represents 100 μm.

**Figure 7:**
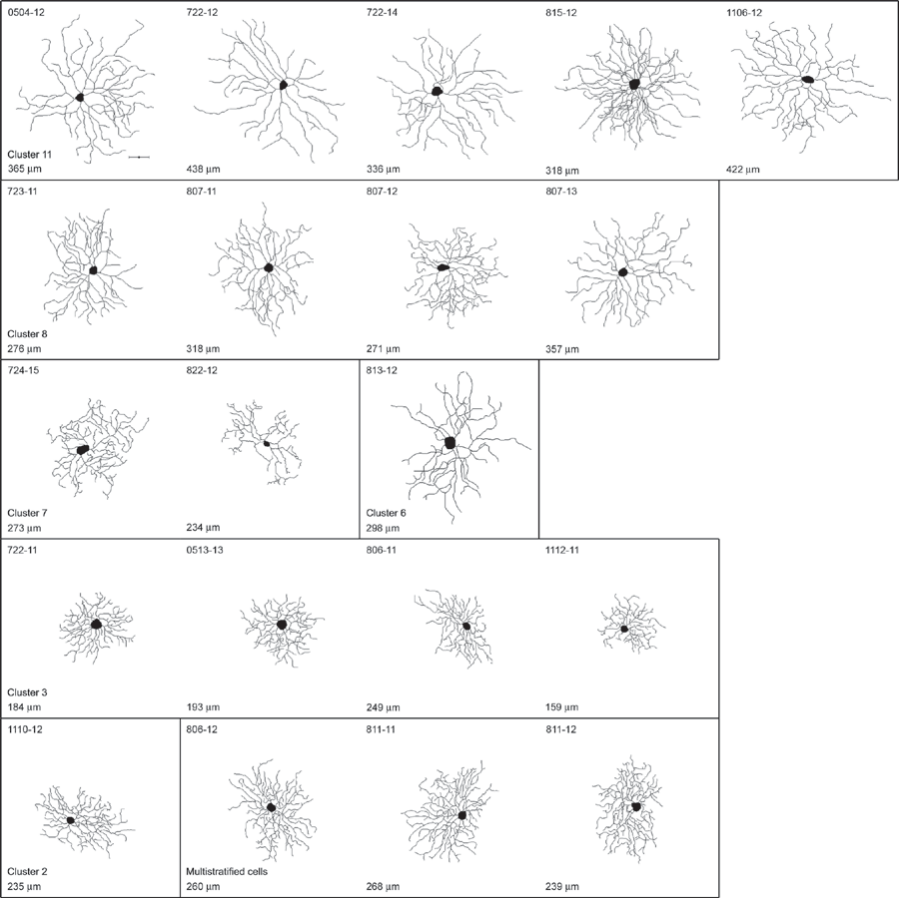
Tracings of 20 ganglion cells with cluster assignment. Neurolucida tracings of ganglion cells from photomicrographs. Cells falling into clusters 11 (5 cells), 8 (4 cells), 7 (2 cells), 6 (1 cell), 3 (4 cells), 2 (1 cell), and bistratified cells (3 cells) are shown. The largest dendritic field diameter is indicated for every cell. The scale bar represents 50 μm.

This was further confirmed by the isolation of morphologically identified ganglion cells. For this purpose, identification of the cells required that the cells be imaged before isolation. Twenty-five well-isolated cells in the GFP-M strain were chosen for imaging, followed by microdissection of the labeled cell and dissociation of the piece of tissue as shown in Figure 5. A gallery of some of the ganglion cells chosen for isolation is shown in Figure 6. The cells were then subjected to the same RT-PCR protocol as described above. In addition, the cells were traced from the images for morphological identification (Figure 7). In 4 cases the pictures were of insufficient quality to trace the cell's morphology from the micrograph. One of the cells was too large to be traced on a single image, but this cell was identified as a photosensitive ganglion cell by the expression of mRNA for melanopsin (see below). From the other 20 cells, we recorded three morphological parameters: dendritic field area, defined as the smallest convex polygon around a cell's dendritic tree, dendritic density, defined as total dendritic length divided by dendritic field area, and stratification depth within the inner plexiform layer [7,26,28]. These parameters were used to compare the cells in this sample with an earlier study from this laboratory [26].

According to this classification, 5 cells in our sample belonged to cluster 11, which is a group of large ON cells, presumably corresponding to the ON-α cells described in various other species (Figure 7). All of these cells expressed NMDA receptor subtype 2d. Four cells fell into cluster 8, another group of relatively large ON-cells, and 3 out of these cells also expressed NR2d.

Two cells were classified as belonging to cluster 7, but one case (cell 822-12) lay almost exactly between clusters 6 and 7. This cell is morphologically distinct from any other in this or in Kong's sample, and may represent a rare type of ganglion cell. Four cells belonged to cluster 3, a group of small OFF-cells. These 4 cells were negative for GluR1, GluR7, and NR2d. Clusters 6 and 2 were represented with one cell each only.

Not included in Kong's classification of mouse ganglion cells were multistratified cells, which are therefore given here as a separate "cluster". Two of these cells were negative for GluR2, and none expressed GluR1.

**Figure 8:**
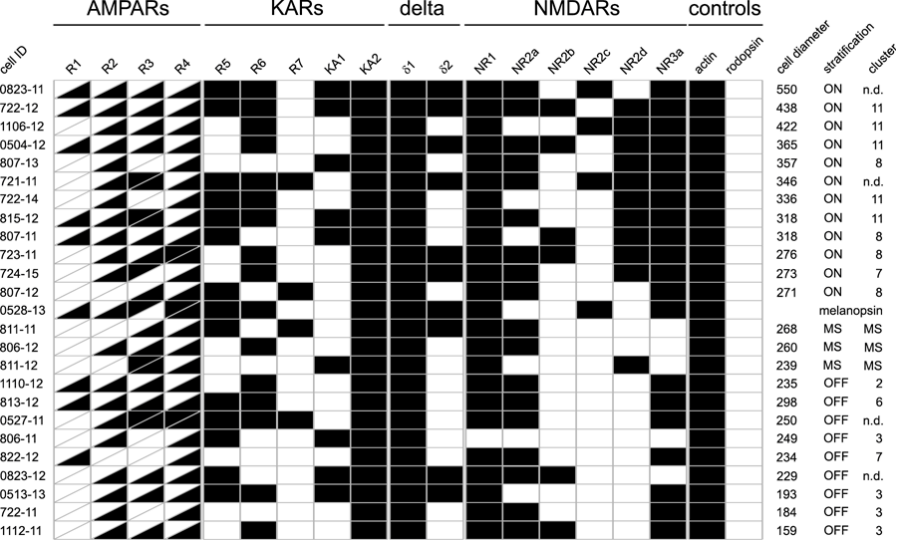
Ionotropic glutamate receptor expression profile in morphologically characterized adult mouse ganglion cells. Cell size, as defined as the largest diameter of the dendritic arbor in mm, stratification ON layer, OFF layer, or multistratified (MS) cells, and cluster designation are also indicated. Some of these cells are shown in Figure 5. n.d. indicates cell could not be traced.

**Figure 9:**
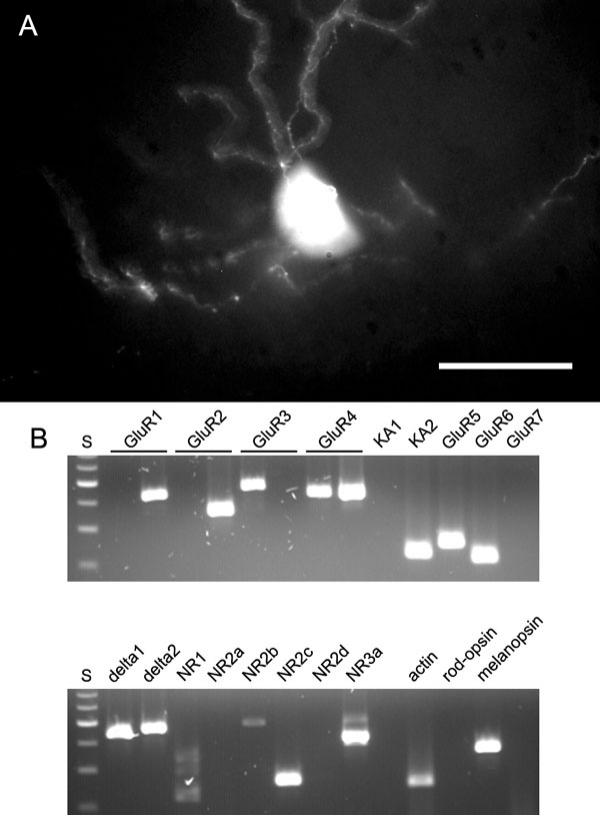
Melanopsin-containing ganglion cell. **A**: The picture shows a projection of 10 image planes taken at 1 mm step size to reveal the dendritic morphology of this melanopsin positive ganglion cell. (Note that this cell was GFP-positive in a GFP-M retina. Therefore the morphology is obvious without immunostaining.) the scale bar represents 50 μm. **B**: Agarose gels showing the iGluR expression in this one cell, which was positive for GluR1flop, GluR2flop, GluR3flip, GluR4flip and flop, KA2, GluR5, GluR6, δ1 and δ2, NR1, NR2c, NR3a. In addition, this cell was assayed for the expression of melanopsin.

To summarize, all cells in this sample expressed at least two isoforms of AMPA receptors, again with the combination GluR2,R3,R4 being the most common All cells expressed KA2, in all but two cases together with other kainate receptor subunits, and 20/21 cells expressed NMDA receptors. Only for two receptor isoforms did we observe differences between groups: NR2d was expressed in 10/12 ON cells, but only in one multistratified cell, and never in small or medium OFF cells. Also, the δ2 subunit was rarely found in OFF cells (2/8) whereas it was detectable in 6 of the ON cells (summarized in Figure 8). Four of the cells in this sample were negative for GluR2. Interestingly, two of them were bistratified cells and may be the mouse equivalent to the ON/OFF directionally sensitive cell described first in rabbits [4]. We observed one cell that was similar to the melanopsin positive ganglion cells recently reported discovered in the rodent retina and others [29-31]. This cell contained AMPA receptors (GluR1,R2,R3,R4), kainate receptors (KA2, GluR5, GluR6), δ1 and δ2, and NMDA receptors (NR1, 2c, 3a), consistent with the observation that these cells, in addition to being intrinsically photosensitive, receive conventional amacrine and bipolar cell input [32], (Figure 9).

### Glutamate receptors of ganglion cells at P5

We examined single ganglion cells from an YFP-12 mouse at postnatal day 5. Though these ganglion cells do not yet show the characteristic morphology, they can be identified by their fluorescence. As expected, the flip isoforms of the AMPA receptors, in particular GluR1 was more commonly observed in immature ganglion cells than it is in the adult cells (Figure 3), but otherwise their glutamate receptor signatures are similar.

### Decision tree for the classification of retinal neurons

**Figure 10:**
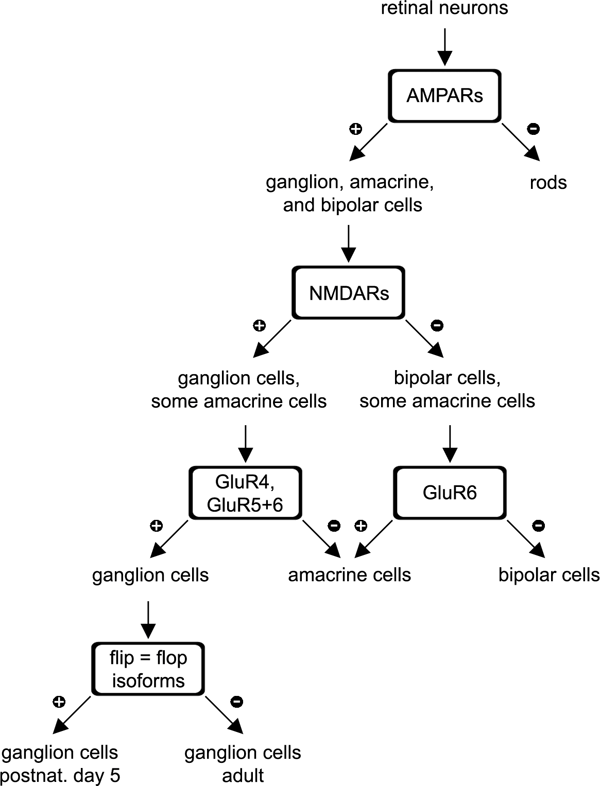
"Decision Tree" structure of iGluR expression in retinal neurons. Retinal neurons can be classified in a binary "decision tree" based on their glutamate receptor expression pattern. Neurons that express only kainate receptors are classified as rod photoreceptors. NMDAR negative cells may be either amacrine or bipolar cells; bipolar preferentially cells express GluR5, and amacrine cells GluR6. The remaining NMDAR positive cells are either ganglion cells or amacrine cells. Ganglion cells almost always express GluR4 and a combination of GluR5-7. P5 ganglion cells express the flip isoforms of the AMPARs almost as commonly as the flop isoforms.

Our data may be summarized by a "decision tree" [33] based on a series of binary decisions (Figure 10). Out of all retinal neurons (excluding cones and horizontal cells, which were not studied), those that express only kainate receptors are classified as rod photoreceptors. NMDAR negative cells may be either amacrine or bipolar cells; the expression of GluR5 in bipolar cells and GluR6 in amacrine cells distinguishes between them. The remaining NMDAR positive cells are either ganglion cells or amacrine cells; the preferential expression of GluR4 and a combination of GluR5-7 in ganglion cells discriminates between ganglion cells and the NMDAR positive amacrine cells. Finally, in immature ganglion cells (P5), the flip isoforms of the AMPARs are expressed almost as commonly as the flop isoforms. In our sample of 97 cells, three bipolar cells (BPC3, CB4a cells 2, and 3) would be mis-sorted as rods, one amacrine cell (AC2) classified as a ganglion cell, another (AC5) cannot be classified, and one ganglion cell (806-11) is classified as a bipolar cell.

## Discussion

Perhaps the main contribution of the present work is the simultaneous evaluation of the whole array of ionotropic glutamate receptors in morphologically identified individual cells. We found that glutamate receptors distinguish between the major cell classes in the mouse retina. In contrast, ganglion cell types are not defined by characteristic patterns of their glutamate receptors. Rather, it is a common feature of ganglion cells to co-express AMPA-, kainate-, and NMDA receptors, usually in multiple isoforms.

This is unlikely to be an artifact due to contamination of the PCR reactions. All cells were washed during the isolation procedure. They were always tested against negative internal (rod-opsin) and external (aspirated medium without cellular contents) controls. Perhaps most important, we see an obvious difference between ganglion cells and other classes of retinal neurons, which were isolated and processed by an identical method. Two restrictions apply to the interpretation of our results: we did not attempt to compare the mRNA levels of individual cells with each other, so we cannot exclude that there may be differences in expression levels between cell types. Also, RT-PCR gives no information as to where in the cell a receptor is expressed. Therefore, we cannot distinguish between dendritic receptors and those that might be expressed as autoreceptors on the axon terminals of ganglion cells.

Our choice to focus upon glutamate receptors was prompted by histological and electrophysiological findings. First, glutamate receptors have been shown to be differentially expressed in the retina by a variety of histological methods. Both by in-situ hybridization and immunostaining, AMPA-, kainate-, and NMDA receptors have been detected in many, but not all cells in the ganglion cell layer [34-37]. Also, the pattern of immunolabeling in the IPL for most receptor subtypes generally show banding patterns, or at least sublayers of more intense staining, that indicate differences in receptor gene expression. Second, though AMPA receptors are involved in the excitatory transmission from bipolar to ganglion cells, the contribution of kainate receptors is less clear (14). NMDA receptors have been identified on ganglion cells, and their localization may be extrasynaptic [38]. Our results are consistent with most of this previous work [15,39]. The multiple receptors shown here to be co-expressed on single cells must act cooperatively to form the final physiological selectivity of the ganglion cells, in ways that are yet to be revealed.

Out of the 97 total cells in our sample, all expressed at least some ionotropic glutamate receptors. The most obviously recognizable group is rod photoreceptors, which are positive for the kainate receptor subtypes KA1, GluR6 and sometimes KA2. These receptors must be autoreceptors, and it is interesting in this context that a recent study has identified GluR6/7 immunoreactivity in fingerlike protrusions from the presynaptic rod terminal into the synaptic cleft in primate retina [40]. Though it is not known whether the mouse rod spherule also has these "fingers," high-affinity kainate autoreceptors are likely to exist in mouse rods as well. In contrast, we found no evidence for the expression of GluR2/3 [41] or NR1 [42].

The bipolar cells in our sample express AMPA (usually R1, sometimes R2, though not R3/4) and/or kainate receptors (preferring KA2 and R5), but we found no expression of NMDA receptors except NR1. As functional NMDA receptors require the co-assembly of NR1 with NR2 or NR3 subunits, these cells are not expected to be NMDA responsive. Recently, AMPA and kainate receptors have been identified on OFF cone bipolar cells where they mediate signal transmission from the cone pedicle to the bipolar cell dendrites and filter the cone signals into distinct temporal channels [27,43]. Though the metabotropic glutamate receptor mGluR6 has been shown to mediate synaptic transmission from the photoreceptor terminals to ON bipolar cells, the AMPA receptor subunit GluR2, and occasionally other AMPA receptor subunits, too, have recently been detected in rod bipolar cells by a RT-PCR approach [44]. The involvement of NMDA receptors in bipolar cell physiology is less clear. Though there are reports of NR2d expression [45] and NR1 [46] at least in some bipolar cells, other studies found little evidence that NMDA evokes currents in bipolar cells [47,48]. Our data also suggest that bipolar cells do not have functional NMDA receptors. However, there are 12 types of bipolar cells, and our sample is very unlikely to include a specimen of every type. Thus, we cannot rule out that there may be one or more types of bipolar cells that do express functional NMDA receptors, but it does not seem to be a common feature.

Amacrine cells are even more diverse than bipolar or ganglion cells [49]. Recordings have identified AMPA-, kainate-, or NMDA- activated currents in several types of amacrine cells [50-52]. Though a survey of glutamate expression in all kinds of amacrine cells is beyond the scope of this paper, we find that all amacrine cells in our sample express AMPA and kainate receptors. In contrast to ganglion cells, GluR1 and -2 is the preferred subunit composition of AMPARs, and in contrast to bipolar cells, amacrine cells seem to prefer GluR6 in addition to KA2 for kainate receptors. In addition, there is at least one population of amacrine cells that does not express NMDA receptors.

Our data suggest that cell classes in the retina can be distinguished according to their glutamate receptor expression profile in a binary decision tree (Figure 10). At the next level of classification-cell types within the five classes of neurons in the retina-glutamate receptor expression itself, in the simple sense studied here, is not informative: there are no clear-cut clusters of subtype expression that coincide with morphological types of ganglion cell. This does not, of course, preclude that differential amounts of the receptor types are expressed, or that they are concentrated differentially at particular synapses. Both of these events could create substantial differences in the receptor mediated electrophysiology of different ganglion cell types.
